# Microbial inoculants for the biocontrol of *Fusarium* spp. in durum wheat

**DOI:** 10.1186/s12866-015-0573-7

**Published:** 2015-10-30

**Authors:** Loredana Baffoni, Francesca Gaggia, Nereida Dalanaj, Antonio Prodi, Paola Nipoti, Annamaria Pisi, Bruno Biavati, Diana Di Gioia

**Affiliations:** Department of Agricultural Sciences, University of Bologna, Viale Fanin 44, 40127 Bologna, Italy; Department of Industrial Chemistry, Faculty of Natural Sciences, Tirana University, Bulevardi Zogu i Parë, Tirana, Albania; Institute of Earth Systems, Division of Rural Sciences & Food Systems, University of Malta, Msida, Malta

**Keywords:** Biocontrol, Lattobacilli, Endophytes, Fusarium head blight

## Abstract

**Background:**

Fusarium head blight (FHB) is a severe disease caused by different *Fusarium* species, which affects a wide range of cereal crops, including wheat. It determines from 10 to 30 % of yield loss in Europe. Chemical fungicides are mainly used to reduce the incidence of FHB, but low environmental impact solutions are looked forward. Applications of soil/rhizobacteria as biocontrol agents against FHB in wheat are described in literature, whereas the potential use of lactobacilli in agriculture has scarcely been explored.

**Results:**

The aim of this work was to study the inhibitory effect of two bacterial strains, *Lactobacillus plantarum* SLG17 and *Bacillus amyloliquefaciens* FLN13, against *Fusarium* spp. *in vitro* and to assess their efficacy in field, coupled to the study of the microbial community profile of wheat seeds. Antimicrobial assays were performed on agar plates and showed that the two antagonistic strains possessed antimicrobial activity against *Fusarium* spp. In the field study, a mixture of the two strains was applied to durum wheat i) weekly from heading until anthesis and ii) at flowering, compared to untreated and fungicide treated plots. The FHB index, combining both disease incidence and disease severity, was used to evaluate the extent of the disease on wheat. A mixture of the two microorganisms, when applied in field from heading until anthesis, was capable of reducing the FHB index. Microbial community profile of seeds was studied via PCR-DGGE, showing the presence of *L. plantarum* SLG17 in wheat seeds and thus underlining an endophytic behavior of the strain.

**Conclusions:**

*L. plantarum* SLG17 and *B. amyloliquefaciens* FLN13, applied as biocontrol agents starting from the heading period until anthesis of wheat plants, are promising agents for the reduction of FHB index.

## Background

Fusarium head blight (FHB) is a severe disease diffused worldwide affecting a wide range of cereal crops such as wheat (*Triticum* L.) and barley (*Hordeum* L.). Only in Europe the loss due to FHB in wheat harvest is estimated from 10 to 30 % [1]. The causal agents are mainly *Fusarium graminearum* Schwabe [teleomorph, *Gibberella zeae* (Schwabe) Petch] and *Fusarium culmorum* (WG Smith) Sacc*.* [2]. These *Fusarium* species are known to produce several mycotoxins, which can accumulate in the grains and are toxic to both animals and humans [1]. FHB epidemics in nature can strike suddenly, but its severity can vary depending on climate conditions, being favored by high humidity and rainfall during flowering [3]. Several authors reported different control measures for FHB [4–6], that include the use of agronomic control techniques, wheat genetic resistance and the use of chemical or biological antagonists. The limited time in which the heads are susceptible to FHB infection (during anthesis and for a short period afterwards) makes this disease a potential target for biological control.

The use of microorganisms against plant pathogens has increased in recent years due to consumers’ demand of a reduced chemical products use for the benefits of human health and the environment [7]. Some successful applications of soil/rhizobacteria as biocontrol agents against FHB, with a reduction of *Fusarium* incidence and mycotoxin level, have already been reported in *in vitro* assays, greenhouse experiments and field trials [6, 8–10]. These bacteria can also colonize root surface and enter root tissues, thus becoming endophytic. Endophytes are of special interest in the field of biocontrol, as they are harmless to plants and are able to inhabit specific ecological niches [11]. From the internal tissues, they can be a source of secondary metabolites acting as elicitors of plant defenses or as antimicrobial agents with potential use to control disease [12]. Typical examples of these molecules are cyclic lipopeptides (LPs) mainly synthesized by species of *Bacillus* and *Pseudomonas* [13]. Nevertheless the array of secondary metabolites produced by endophytic bacteria remains to be discovered [14].

In addition to these strains, *Lactobacillus* spp. members can be interesting as potential biocontrol agents. They are well known as probiotics and protective microorganisms in food and feed against spoilage bacteria or fungi [15]. However, in the agricultural field, their potential use is still sparse, although they can be found inside plant tissues [16, 17] and produce bioactive compounds (organic acids, bacteriocins, phenyllactic acid, cyclic dipeptides, fatty acids) with antimicrobial properties against a broad spectrum of phytopathogenic fungi. In particular, different molecules are active against *Fusarium* [18–20] and some *Lactobacillus* strains have been found to be capable of mycotoxin detoxification [21, 22].

In this work, the inhibitory effect of *Lactobacillus plantarum* SLG17 and *Bacillus amyloliquefaciens* FNL13 was evaluated against strains of *F. culmorum* and *F. graminearum* isolated from wheat, and the presence of genes responsible for antimicrobial peptide production was checked. Furthermore, the antagonistic efficacy in controlling FHB was studied in field conditions. PCR-DGGE analyses on wheat seeds were performed to investigate: a) the microbial community profile of wheat grains upon different treatments; b) the endophytic colonization feature of the microbial inoculants; c) *Fusarium* population in wheat.

## Results

### Antimicrobial activity against *Fusarium* spp

**Fig. 1:**
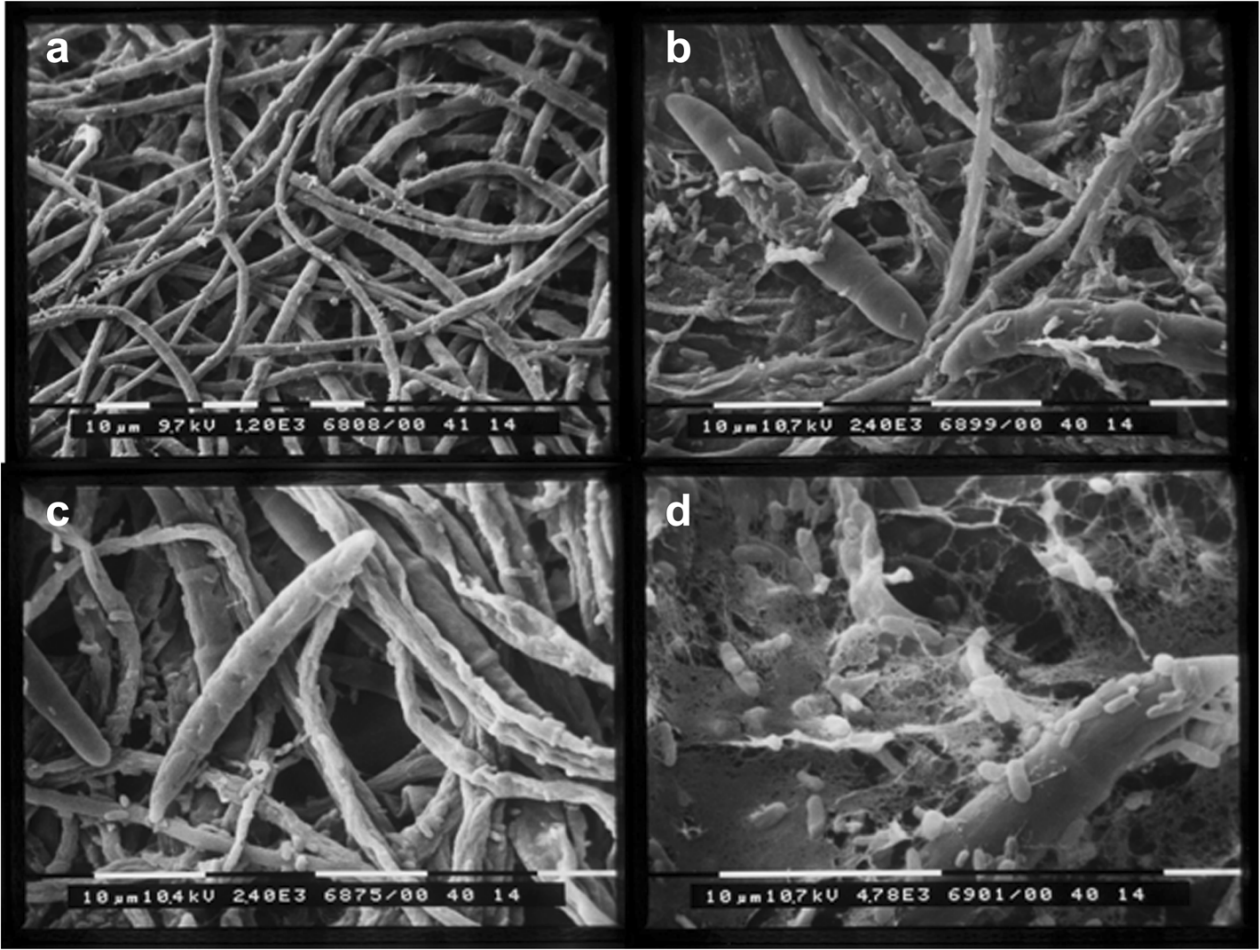
SEM analysis of antagonistic *B. amyloliquefaciens* FLN13 interacting with hyphae of *F. culmorum* Fc1 on PDA medium at 5th day after incubation. **a** normal hyphae of *F. culmorum* Fc1; **b**, **c** damaged hyphae and macroconidia of *F. culmorum* Fc1; **d** damaged hyphae with cells of *B. amyloliquefaciens* FLN13 embedded in a extracellular matrix

*In vitro* tests performed on agar plates showed that the two strains used in this work, *L. plantarum* SLG17 and B. *amyloliquefaciens* FLN13, possessed antimicrobial activity against all the five *Fusarium* strains isolated from diseased wheat (Table 1). Both antagonistic strains did not display reciprocal inhibition (data not shown). Antimicrobial activity of *B. amyloliquefaciens* FNL13 against *F. culmorum* Fc1 was also examined using scanning electron microscopy (SEM) since hyphae adjacent to the inhibition area in the plate tests revealed an unusual brown color. The adherence of *B. amyloliquefaciens* FNL13 cells on the hyphal surface of *F. culmorum* Fc1 is evident, and the contact zone showed shrinkage with loss of turgidness of hyphae (Fig. 1b) compared to the control grown hyphae (Fig. 1a). Conidia were also subjected to surface alteration (Fig. 1c). In addition, as shown in Fig. 1d, bacterial aggregates close to hyphae embedded in an extracellular matrix could be observed (Fig. 1c).

**Table 1:** Source of isolation, species identity and fungal inhibition spectra of *L. plantarum* SLG17 and *B. amyloliquefaciens* FLN13; values are expressed as mean ± SD

	Antifungal activity: inhibition radius (mm)					
Isolates	*F. culmorum* Fc1	*F. culmorum* Fc2	*F. culmorum* Fc3	*F. graminearum* Fg1	*F. graminearum* Fg2	Source
*L. plantarum* SLG17	13.67 ± 0.58	12.67 ± 2.08	17.33 ± 2.31	15.67 ± 1.15	16.33 ± 3.21	Silage
*B amyloliquefaciens* FLN13	10.87 ± 0.06	10.80 ± 0.10	10.67 ± 0.21	10.43 ± 0.06	10.67 ± 0.06	Forest soil

### Detection of genes for plantaricin and LPs synthesis

*L. plantarum* SLG17 was positive for *pln*E/F, *pln*K, *pln*G, *pln*N genes, whereas the *pln*J gene was not detected (data not shown). The PCR assay using degenerated primers followed by cloning and sequencing of the amplified bands allowed the detection of the genes related to the biosynthesis of surfactin, fengycin and mycosubtilin in the genome of *B. amyloliquefaciens* FLN13 (Table 2). On the other hand, the *ituC* fragment, involved in iturin A production, was not detected.

**Table 2:** Blast results of the sequenced products obtained from PCR amplification using gene-specific degenerated primers from biosynthetic genes of Mycosubtilin synthase, Fengycin synthetase and Surfactin synthase in *B. amyloliquefaciens* FLN13

Accession Number (GenBank)	Primer name	Size (bp)	GeneBank accession number	Identity (%)	*E* value	UniProt Accession number
KP944004	Am1-F/Tm1-R	405	HG328254.1	99 %	0.0	BmyB protein, Mycosubtilin synthase subunit B (S6FI96)
KP944005	Af2-F/Tf1-R	441	HF563562.1	99 %	0.0	FenD protein, Fengycin synthetase (M1XBL6)
KP944006	As1-F/Ts2-R	420	CP003838.1	98 %	0.0	SrfAB protein, Surfactin synthase subunit 2 (L0BHY7)

### In field trial and FHB assessment in wheat

The isolation of FHB agents in the wheat grains resulted positive (75 % of kernel were positive to *Fusarium* spp. in the Ctr), confirming natural infection of the field.

DI was not significantly different among the four treatments at GS 73, whereas a few differences were observed in DS, with the highest values present in Ctr and Bio-2 (9.2 % and 11.5 %) and the lowest in Chem (3.3 %). Regarding the FHB index, the lowest value was obtained in Chem (1.6), being statistically different from all the other treatments (Table 3).

**Table 3:** Mean values of FHB incidence (DI), disease severity (DS) and FHB index at Zadoks growth stage GS 73 and GS 87 in the different treatments (Ctr, Chem, Bio-1, Bio-2)

Treatment*	GS 73			GS 87		
	DI (%)	DS (%)	FHB index	DI (%)	DS (%)	FHB index
Ctr	30.0^a^	9.2^ab^	2.8^b^	51.4^b^	17.5^b^	9.1^c^
Chem	18.0^a^	3.3^a^	0.5^a^	30.5^a^	5.5^a^	1.6^a^
Bio-1	23.0^a^	7.2^ab^	1.6^b^	36.0^a^	13.0^ab^	4.6^ab^
Bio-2	29.0^a^	11.5^b^	3.5^b^	54.0^b^	17.4^b^	9.0^bc^

*Within columns, means followed by different letters differ significantly (Tukey’s test, *P* ≤ 0.05)

In the evaluation made at GS 87, the highest DI, DS and FHB index were observed in Ctr (51.4 %, 17.5 % and 9.1) and Bio-2 (54 %, 17.4 % and 9.0) and no significant differences were observed between them. On the other hand, a significant FHB index reduction was obtained in Bio-1 (49.5 % reduction) and Chem (82.4 % reduction) compared to Ctr; in both Bio-1 and Chem the DI is statistically different with respect to Ctr (36 % and 30.5 % vs 51.4 %), whereas DS showed a significant reduction only in Chem (5.5 % vs 17.5 %). The kernel weight was significantly increased (*P* ≤ 0.05) only in Chem (average weight: 2814 ± 99 g); on the other hand, the observed increase in BIO-1 was not significant compared to Ctr (2518 ± 99 g vs 2455 ± 102 g). Bio-2 had a kernel weight lower than Ctr (2410 ± 79) (Table 3).

### PCR-DGGE analysis

**Fig. 2:**
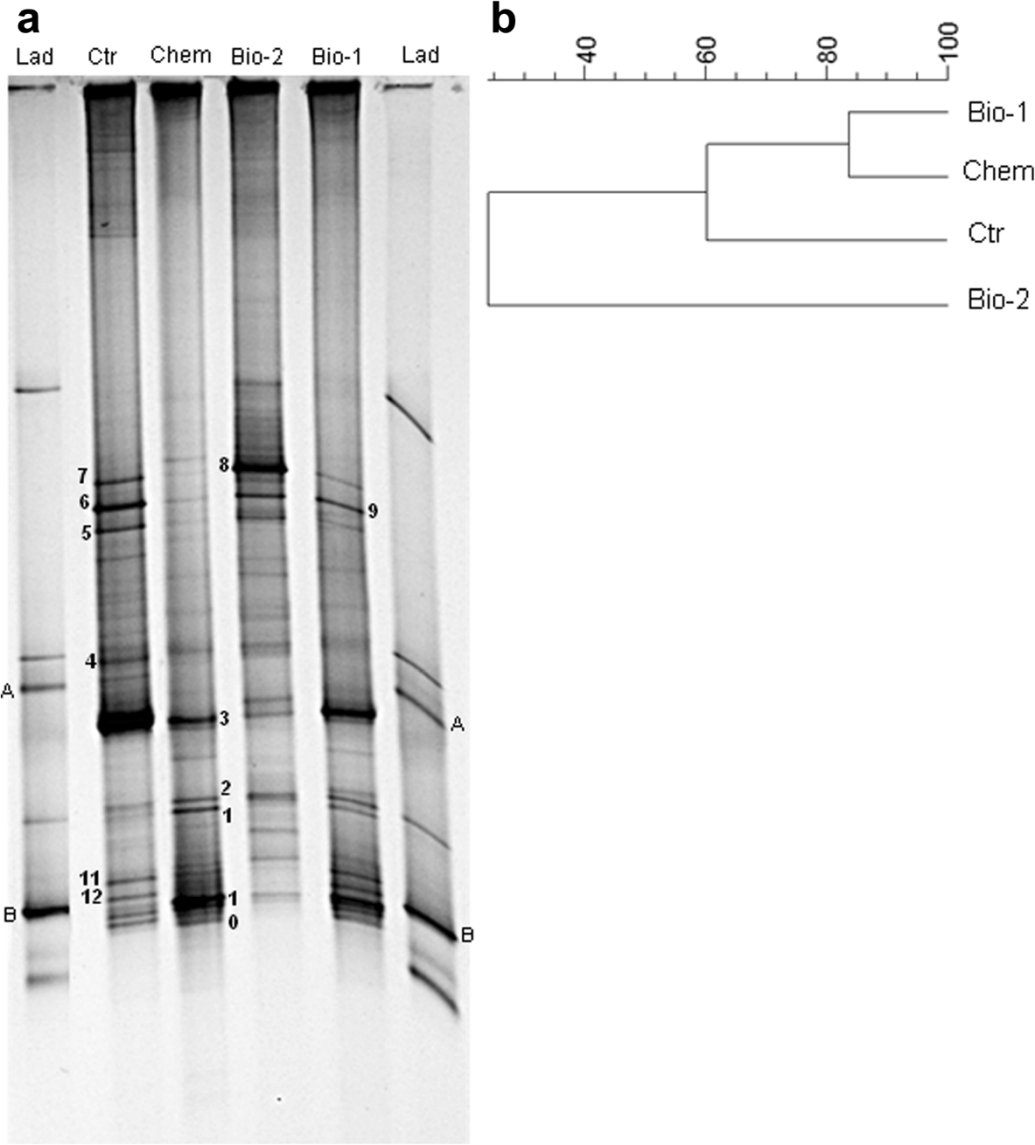
DGGE patterns of eubacterial 16S rDNA fragments amplified from wheat seeds (**a**) and cluster analysis (**b**); Ctr: untreated subplots; Chem: prothioconazole treated subplots; Bio-1: subplots treated with *L. plantarum* SLG17 and *B. amyloliquefaciens* FLN13 (weekly treatment-at Zadoks growth stage 50 to 67); Bio-2: subplots treated twice with L. plantarum SLG17 and *B. amyloliquefaciens* FLN13 at Zadoks growth stage 67 and 70; Lad: Ladder with known microorganism. A: *L. plantarum* SLG17; B: *B. amyloliquefaciens* FLN13

Wheat seed bacterial community was firstly investigated in all treatment condition by PCR-DGGE analysis of 16S rRNA gene fragments targeting total eubacteria. Profiles of the bacterial communities and UPGMA dendrogram are shown in Fig. 2a and b. The cluster analysis showed a distinct division between Bio-2 and the group Ctr, Chem and Bio-1 (similarity less than 27 %), and a further division distinguishes Ctr from Chem and Bio-1 profiles.

**Fig. 3:**
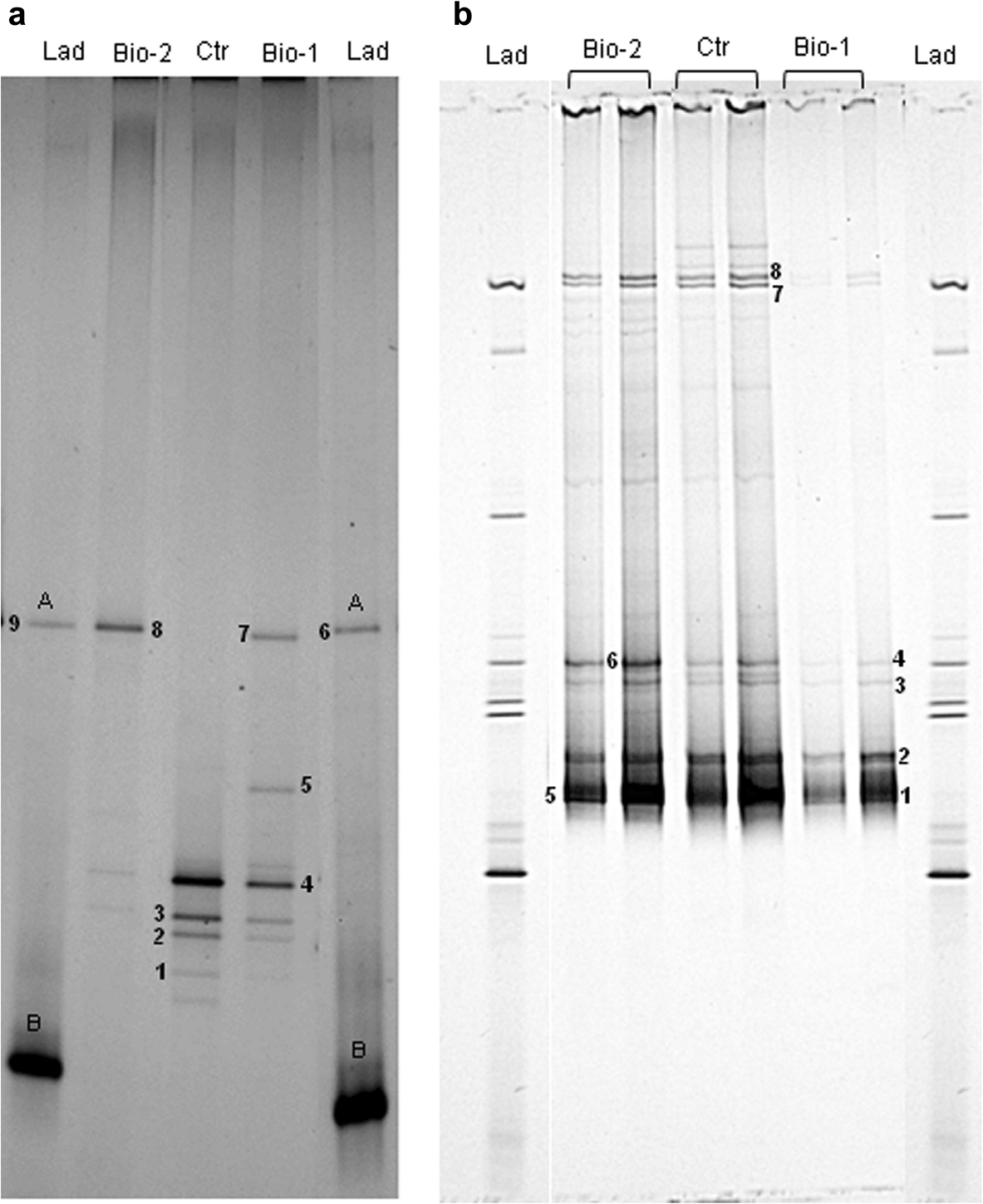
DGGE patterns of lactobacilli 16S rDNA fragments from wheat seeds (**a**) and DGGE patterns of partial region of the translation elongation factor1 alpha (EF-1 alpha) gene of *Fusarium* spp. from wheat seeds (**b**); Ctr: untreated subplots; Chem: prothioconazole treated subplots; Bio-1: subplots treated with *L. plantarum* SLG17 and *B. amyloliquefaciens* FLN13 (weekly treatment-at Zadoks growth stage 50 to 67); Bio-2: subplots treated twice with L. plantarum SLG17 and *B. amyloliquefaciens* FLN13 at Zadoks growth stage 67 and 70; Lad: Ladder with known microorganism. A: *L. plantarum* SLG17; B: *B. amyloliquefaciens* FLN13. Numbers indicate excised and sequenced DGGE bands

Sequencing of selected bands evidenced the presence of five different bacterial species (Table 4). Band 1, detected in Chem and Bio-1, showed 100 % similarity to *Lysobacter soli*, while bands 2, 3, and 12 (detected in all profiles, except for band 12 which is present only in Ctr and Bio-1) had 100 % similarity to *Pantoea agglomerans.* Bands 4, 5 and 7 showed 100 % identity with *Erwinia rhapontici*; bands 6, 9 (detected in all profiles) and 8 (Bio-2) were identified as *Rahnella acquatilis* (100 % identity). DGGE fingerprints targeting lactobacilli were rather simple, with a few well-defined bands (Fig. 3a), not detectable in all samples. Bands 1, 2, 3, 4 were particularly visible in the Ctr profile while their intensity decreased in the profiles Bio-1 and Bio-2. These bands and band 5 (Bio-1) had 100 % identity with *Exiguobacterium sibiricum* (Table 4). Bands 7 and 8 were detected only in Bio-1 and Bio-2 profile, and had the same migration distance of bands 6 and 9, which represent *L. plantarum* in the ladder profile. These four bands were also excised and sequenced, showing 100 % similarity with *L. plantarum* (Table 5). Unexpectedly, primers targeting lactobacilli allowed the amplification of *B. amyloliquefaciens* FLN13, which was thus present in this DGGE profile. An *in silico* evaluation of Lac1 and Lac2 primer sequences on 16S rDNA of *Bacillus* spp. evidenced a possible amplification of the ribosomal DNA.

**Table 4:** Eubacteria sequence alignment with blast

Band	Closest match	% similarity^a^	Accession number (GenBank)
1	*Lysobacter soli - L. enzymogenes*	100 %	KP943989
2	*Pantoea agglomerans*	100 %	KP943990
3	nd	-	-
4	*Erwinia rhapontici strain*	100 %	KP943991
5	*Erwinia rhapontici strain*	100 %	KP943992
6	*Rahnella aquatilis* strain	100 %	KP943993
7	*Erwinia rhapontici strain*	100 %	KP943994
8	*Rahnella aquatilis* strain	100 %	KP943995
9	nd.	-	-
10	nd	-	-
11	nd	-	-
12	*Pantoea agglomerans*	100 %	KP943996

^a^Similarity represents the % similarity shared with the sequences in the GenBank database. nd: not determined

**Table 5:** Lactobacilli sequence alignment with blast

Band	Closest match	% similarity^a^	Accession number
1-2-3	*Exiguobacterium sibiricum*	99 %	KP943997
4	*Exiguobacterium sibiricum*	100 %	KP943998
5	nd	-	-
6-9	*Lactobacillus plantarum*	100 %	KP943999
7–8	*Lactobacillus plantarum*	100 %	KP944000

^a^Similarity represents the % similarity shared with the sequences in the GenBank database. nd: not determined

The DGGE fingerprints of the *Fusarium* community were also simple and not many differences were detected among plots treated with microorganisms and the control plots (Fig. 3b). A lower band intensity was shown in Bio-1 profile. Mainly, bands excised (1, 2, 3 and 4) had 100 % similarity to *F. culmorum* (Table 6) and were detected in all profiles. Bands 7 and 8, whose detection was weak in Bio-1, had sequence homology with *F. graminearum*.

**Table 6:** *Fusarium* sequence alignment with blast

Band	Closest match	% similarity^a^	Accession number
1-2-5	*Fusarium culmorum*	100 %	KP944003
3-4-6	*Fusarium culmorum*	100 %	KP944002
7-8	*Fusarium graminearum* strain	100 %	KP944001

^a^Similarity represents the % similarity shared with the sequences in the GenBank database

## Discussion

The use of microorganisms for pathogen control in plants is largely studied and applied to several crops of economic value, with consequent reduction of the spread of chemical agents in the environment. *Bacillus* spp. are the most commercially successful biocontrol agents, with more than 10 *Bacillus*-based registered formulations [23], whereas *Lactobacillus* spp. strains, although included in multi-strain bio-fertilizers [17], have rarely been used for biocontrol intervention strategy [24]. In this work, two bacterial strains, *B. amyloliquefaciens* FLN13 and *L. plantarum* SLG17, previously selected for their strong inhibitory activity against several plant pathogenic fungi, were checked for their *in vitro* antifungal activity against strains of *F. culmorum* and *F. graminearum* and, as a mixture, they were tested for their potential to control FHB in durum wheat in field.

*L. plantarum* SLG17 exhibited antimicrobial activity against the tested *Fusarium* strains, and a consistent number of genes responsible for plantaricin production. Beside the role of organic acids which are known to be detrimental for pathogen growth [15], a possible action of plantaricins in the antagonist activity against FHB agents can be suggested. Moreover, results obtained from DGGE profiles of seeds deriving from wheat subjected to both biological treatments (Bio-1 and Bio-2) demonstrated that *L. plantarum* SLG17 was able to enter the internal tissues of spikelets to become an endophyte. Although no direct evidence (e.g. microscopic examination) was given to show the penetration of the sprayed microorganism, the absence of bands ascribed to *L. plantarum* in the Ctr profile strengthen the hyphotesis of the endophytic behavior of *L. plantarum* SLG17. Moreover, no data on *Lactobacillus* detection on wheat seeds are at present available in the literature, unless in silage fermentation [25]. In addition, *B. amyloliquefaciens* FLN13 was also able to inhibit *in vitro* all tested *Fusarium* strains and possessed the synthetase genes responsible for mycosubtilin, fengycin and surfactin production, which are known to be among the molecules (LPs, polyketides, antibiotics) involved in the antagonistic activity against plant pathogens [13]. In the *in vitro* antagonistic assay, morphological changes were observed by SEM analyses in the hyphae and macroconidia of *F. culmorum* Fc1, associated with the presence of an extracellular matrix (Fig. 1). These effects on fungi have already been described by several authors in the presence of different species of *Bacillus*, as a result of cell wall degradation, increased cell adhesion and lipopeptide excretion [8, 26, 27]. In addition, Gong et al. [28] clearly showed the destructive effect of secondary metabolites (iturin A and plipastatin A) from *B. amyloliquefaciens* S76-3 in *F. graminearum* conidia and hyphae. Unfortunately, no evidence on the presence of this strain inside wheat seeds was found in the PCR-DGGE profiles. As also reported by Crane et al. [29], following application of *B. amyloliquefaciens* strain TrigoCor on wheat, most *Bacillus* cells were present post-application as metabolically dormant spores, thus hindering potential internalization in the host plant. It should also be emphasised that the detection limit for DGGE is in the order of 10^3^ cfu/ml [30]; as a consequence, microbial groups that are present and active, but whose population is lower than 10^3^ cfu/ml, will not be evidenced.

Beside the clear efficacy of the fungicide treatment, the field trial showed, at GS 87, a significant FHB index reduction (49.6 %) in Bio-1 compared to the untreated subplots. Therefore, the efficacy of a preventive and repetitive treatment with the applied mixture of microorganisms was demonstrated, although this was not associated with a significant increase in kernel yield. On the other hand, the treatment applied twice at flowering (Bio-2) was not effective. This last result is different from what obtained by Schisler et al. [31] who showed a significant DS and DI reduction by using a *B. subtilis/amyloliquefaciens* strain at flowering in a field trial. However, as pointed out by the same author and by Crane et al. [29], several factors can contribute to the successful application of FHB antagonists in the field, such as the decline of antagonistic molecule levels on wheat surface within a few days, the deleterious effects of ultraviolet light, available nutrients, host variety and runoff from rainfall and irrigation. Last but not least, the intrinsic properties of the applied microorganisms and the inoculation technology should also be considered.

The identification of excised bands from the DGGE profiles evidenced the detection of different microbial species (*P. agglomerans, E. raphontici*), commonly isolated from plant tissues [32, 33] and the presence of *F. culmorum* and *F. graminearum*, confirming their endemic presence in wheat kernels. Finally, the PCR-DGGE, targeting lactobacilli, allowed the detection of *L. plantarum* in Bio-1 and Bio-2 seeds, indicating that the inoculated strain was able to become endophyte. This observation confirms that endophytes may not only derive from the rizosphere, but, as reviewed by Compant et al. [34], they may also originate from other sources, such as the phyllosphere, the anthosphere, or the spermosphere.

## Conclusions

The obtained results suggest a possible use of the two microorganisms in controlling FHB spread throughout all the heading and ripening period, aimed at protecting the spikelets before and during anthesis. Moreover, the endophytic colonization of *L. plantarum*, which is one of the most characterized and used probiotic *Lactobacillus* species, can help to increase the nutritional properties of wheat and derived products (silages, flour and related bakery products). Further studies are necessary to deeply investigate the interaction with the host plant, the metabolite production of both strains and improve the inoculation technology.

## Methods

### Microorganisms and culture growth conditions

This work is based on the use of two microorganisms, *L. plantarum* SLG17, isolated from silages and *B. amyloliquefaciens* FNL13, isolated from a forest soil, both selected after an *in vitro* screening aimed at studying the capability of several *Lactobacillus* and *Bacillus* strains of inhibiting the growth of plant pathogenic fungi, including some *Fusarium* species [35]. These two strains, showing the highest antimicrobial activity, were therefore chosen to perform the current experiments with wheat-isolated strains of *F. culmorum* and *F. graminearum,* specifically associated to FHB.

*L. plantarum* SLG17 was grown on de Man, Rogosa and Sharpe (MRS) broth (BD, Difco, USA) for 24 h at 37 °C in anaerobic conditions (1.5 % agar for agar plates); while *B. amyloliquefaciens* FNL13 was grown in Nutrient Broth (NB, BD) for 24 h at 25–28 °C under gentle shaking. For long-term storage, microbial strains were stored at −80 °C.

*F. culmorum* Fc1, Fc2, Fc3 and *F. graminearum* Fg1, Fg2 strains, belonging to the main species involved in FHB, were isolated from naturally infected durum wheat kernels (cv Normanno) from fields at the University Farm of Bologna, located in Cadriano (Bologna, Italy) during 2010–2011 and identified by PCR using species-specific primers [36] (unpublished results). They were cultivated on Potato Dextrose agar plates (PDA, BD). For long-term storage the fungal strains were stored at 4 °C in the fungal collection of the Department of Agricultural Sciences, University of Bologna.

### Assessment of the antimicrobial activity against *Fusarium* spp

#### Conidia production

*Fusarium* macroconidia inocula were obtained by growing the fungal strains on PDA plates for 7 days (or until sporulation). Then conidia were collected gently scraping the mycelium with a scalpel, filtered in cheese cloth, put into slants with sterile peptone water (0.2 % w/v) and the slants were vigorously shaked. Macroconidia concentrations were determined using a haemocytometer, and adjusted to 10^5^ macroconidia per ml of sterile peptone water (0.2 % w/v).

#### Antimicrobial assay

The assay was performed according to Magnusson et al. [19]. 50 μl of *L. plantarum* and *B. amyloliquefaciens* overnight cultures were spotted on MRS and PDA plates, respectively, and allowed to grow for 24 h at 37 °C in anaerobic conditions the former and 25 °C in aerobiosis the latter. The plates were then overlaid with 10 ml of PD soft agar (0.8 %; Difco Laboratories) containing 10^5^*Fusarium* spores (macroconidia) per ml. After 48–36 h of aerobic incubation at 25 °C, the inhibition radius (expressed in mm) was measured. Each assay was performed in triplicate and mean ± SD was recorded. The antimicrobial assay was also performed between the two antagonistic strains.

### SEM analyses

An agar square (1x1 cm) plus mycelium was taken from the inhibition area of the dual cultures *B. amyloliquefaciens* FNL13—*F. culmorum* Fc1. Sample was immersed in 2.5 % glutaraldehyde for 4 h at room temperature, rinsed four times with phosphate buffer (0.1 M; pH 7.2), and dehydratated in a graded series of ethanol concentrations (10, 30, 50, 75, 95 and 100 % for 15 min each). It was then dried with a Critical Point Dryer unit (Balzers CPD 020), mounted on aluminium stub with double-sided tape and coated with gold-palladium film using an ion sputtering unit (Balzers MED 010). The sample was observed under a Philips 515 SEM at 7 KV and photographed with Nikon Coolpix 5400.

### Detection of genes involved in antagonistic activity

The presence of genes belonging to *pln* locus (*pln*G, *pln*E/F, *pln*J, *pln*K, *pln*N) and involved in plantaricin production was investigated according to Doulgeraki et al. [37] on *L. plantarum* SLG17, following DNA extraction from pure culture. In addition, the presence of four families of lipopeptides synthetase genes (surfactin synthetase, fengycin synthetase, mycosubtilin synthetase and IturinA) were also investigated in *Bacillus amyloliquefaciens* FLN13. PCR protocols and cloning procedure were performed according to Tapi et al. [38], while iturinA was analyzed according to Alvarez et al. [39] by amplifying the *Itu*C open reading frame. Primers used are listed in Table 7.

**Table 7:** List of primers for detection of genes belongs to *pln* locus and LPs shyntetase

Target gene	Primers	Annealing temperature (°C)	References
*pln*E/F	fw: 5′- GGCATAGTTAAAATTCCCCCC-3′	53.2	Doulgeraki et al., 2013 [37]
	rev: 5′- CAGGTTGCCGCAAAAAAAG-3′		
*pln*J	fw: 5′-TAACGACGGATTGCTCTG-3′	51	Doulgeraki et al., 2013 [37]
	rev: 5′- AATCAAGAAATTATCACATTAGTC-3′		
*pln*K	fw: 5- CTGTAAGCATTGCTAACCAATC	52.9	Doulgeraki et al., 2013 [37]
	rev: 5- ACTGCTGACGCTGAAAAG		
*pln*G	fw: 5- TGCGGTTATCAGTATGTCAAAG	52.8	Doulgeraki et al., 2013 [37]
	rev: 5-CCTCGAAACAATTTCCCCC		
*pln*N	fw: 5- ATTGCCGGGTTAGGTATCG	51.9	Doulgeraki et al., 2013 [37]
	rev: 5- CCTAAACCATGCCATGCAC		
Surfactin synthetase	As1-f CGCGGMTACCGVATYGAGC	43 °C	Tapi et al., 2010 [38]
	Ts2-r ATBCCTTTBTWDGAATGTCCGCC		
Fengycin synthetase	Af2-f GAATAYMTCGGMCGTMTKGA	45 °C	Tapi et al., 2010 [38]
	Tf1-r GCTTTWADKGAATSBCCGCC		
Mycosubtilins syntetase	Am1-f CAKCARGTSAAAATYCGMGG	45 °C	Tapi et al., 2010 [38]
	Tm1-r CCDASATCAAARAADTTATC		
Iturin A synthetase	ituC-f AAAGGATCCAAGCGTGCCTTTTACGGGAAA	56 °C	Alvarez et al. 2011 [39]
	ituC-r AAAAAGCTTAATGACGCCAGCTTTCTCTT		

### Field trial

#### Experimental trial

The durum wheat cultivar Normanno, susceptible to FHB agents, was sown in autumn 2012 at the University Farm of Bologna, where durum wheat cultivation is repeated every year. The field was subdivided into small subplots (1 m x 2.2 m), one subplot represented one replication, and four replications were used for each treatment. Infection of heads by *Fusarium* spp. was dependent on naturally occurring levels of inoculum and rainfall (20 rainfall events for a total of 5.85 cm of rain, from flowering until ripening, were observed). The wheat stage of growth was measured using the Zadoks growth stage (GS) [40].

Four different treatments were designed: untreated subplots (Ctr), subplots treated with a prothioconazole-based fungicide (Proline®, Bayer Crop Science, Italy), according to the manufacturer instructions (Chem), subplots weekly treated with a suspension of the two microorganisms from heading (GS 50) to the beginning of flowering (GS 67) (Bio-1), subplots treated twice with the microorganisms suspension at GS 67 and at mid-flowering (GS 70) (Bio-2).

#### Production of antagonist inoculum

*L. plantarum* SLG17 and *B. amyloliquefaciens* FNL13 were grown in MRS broth and NB (under shaken), respectively, for 24 h as previously described. 10 ml of the overnight cultures were used as inoculum in flasks containing 400 ml of broth (MRS and NB) for 48 h to reach a concentration of approximately 10^7^ cfu/ml. The cultures were then centrifuged (20 min at 12.000 rpm) and the pellets were combined in 400 ml of sterilized saline solution (0.8 % NaCl). The microbial inoculum was prepared before each treatment and sprayed immediately on the spikelets on late afternoon (100 ml/subplot).

#### Assessment of FHB

Five groups of ten spikes per subplot were chosen and marked with plastic labels for FHB assessment: disease incidence (DI) and disease severity (DS). The disease evaluation was assessed twice at GS 73 and 87. DI was calculated as the percentage of ears that are visibly diseased in relation to the total number assessed (50 spikes/subplot). For DS evaluation, the scale rating of Purahong et al. [41] was applied. This scale represents eight levels of percentage area infected on individual ears: 0 % (no infection), 2 %, 5 %, 10 %, 25 %, 50 %, 75 % and 90 % (90 % or more). DS was calculated as the weighted average of percentage area infected on individual ear in relation to the number of infected ears. Mean FHB Index was calculated as the product of DI and DS, divided by 100 [3]. Kernel yield per treatment was evaluated by calculating the average (±SD) of the kernel weight obtained from each subplot after harvest. The isolation of *F. culmorum* and *F. gramineraum* from 100 wheat grains, harvested from each plot, was performed to verify their presence.

### Statistical analysis

The analysis of variance (ANOVA) was conducted on percentage values subjected to angular transformation for DI, DS and FHB index at both evaluation times. Significant differences among treatments were evaluated using the post hoc Tukey’s test with STATGRAPHICS PLUS software (STATPOINT TECHNOLOGIES, INC., Virginia). The level of significance was set at *P* ≤ 0.05.

### DNA extraction

Wheat seeds from each treatment were collected two weeks before harvesting, and kept at 4 °C until further processing. Before DNA extraction, they were surface-sterilized with 70 % ethanol (3 min), treated with 2 % sodium hypochlorite (NaClO) (5 min), followed by repeated washing with sterile distilled water (3 times for 1 min). An aliquots of the final rinse was plated on Nutrient Agar and Luria-Bertani plates to ensure the surface sterilization efficiency. Later, they were snap frozen in liquid nitrogen and ground by mortar and pestle. Approximately 100 mg of powder was directly used for DNA extraction by applying the DNeasy plant mini kit (Qiagen GmbH, Milano, Italy), according to the manufacturer’s instructions. The purity and concentration of extracted DNA were determined by measuring the ratio of the absorbance at 260 and 280 nm (Infinite 200 PRO NanoQuant, Tecan, Mannedorf, Switzerland).

### PCR amplification of 16S rDNA sequences and elongation factor1*alpha*

Three different PCR amplifications were performed, before DGGE, targeting eubacteria, lactobacilli and *Fusarium* spp. To avoid the amplification of the chloroplast or mitochondrial DNA, a nested PCR approach was used for the detection of eubacteria, according to Chelius and Triplett [42]. Firstly, a PCR was performed using universal primers 799f (5′-AACMGGATTAGATACCCKG-3′) and 1492r (5′-TACGGYTACCTTGTTACGACTT-3′). Following excision of the smaller PCR product (680 pb), a second PCR was then carried out in 30 μl volume, using GC-clamp 968f (5′-CGCCCGGGGCGCGCCCCGGGCGGGGCGGGGGCACGGGGGGAACGCGAAGAACCTTAC-3′) and 1348r (5′-CGGTGTGTACAAGGCCCGGGAACG-3′). To deeply analyze possible differences between the treated plots with microorganisms and the Ctr plots, a PCR-DGGE targeting lactobacilli were also performed with primers Lac1 (5′-AGCAGTAGGGAATCTTCCA-3′) and Lac2-GC (5′-CGCCCGGGGCGCGCCCCGGGCGGCCCGGGGGCACCGGGGGATTYCACCGCTACACATG-3′) according to Gaggia et al. [17].

In addition, amplification of a partial region of the translation elongation factor1 alpha (EF-1α) gene was performed to investigate *Fusarium* diversity, according to Yergeau et al. [43], using the primer couple Alfie1-GC-f (5′-CGCCCGCCGCGCGCGGCGGGCGGGGCGGGGGCACGGGGGGTCGTCATCGGCCACGTCGACTC-3′) and Alfie2-r (5′-CCTTACCGAGCTCRGCGGCTTC-3′).

### Electrophoretic conditions and identification of bands

The DGGE analysis was basically performed as first described by Muyzer et al. [44], using a DCode System apparatus (Bio-Rad, Richmond, CA, USA), employing 7 % polyacrylamide gels with a denaturing range of 35–55 % for total eubacteria, 30–55 % for group-specific lactobacilli and 40–55 % for fungi. Electrophoresis was performed at 65 V for 16 h at 60 °C. Gels were stained in a solution of 1X SYBR-Green (Sigma–Aldrich) in 1X TAE for 20 min and their images captured in UV transillumination with Gel DocTM 226 XR apparatus (Bio-Rad). Similarities between the DGGE profiles were determined by calculating similarity indices of the densitometric curves of the profiles using Pearson correlation coefficient with the aid of computer software (Gel Compare II, Applied Maths, Keistraat, Belgium). An unweighted pair group method using an arithmetic averages (UPGMA) algorithm was performed.

Well-defined bands in all DGGE experiments were excised from the gel with a sterile scalpel and DNA was eluted by incubating the gel fragments for 16 h in 50 μl of sterile deionised water at 4 °C. Two μl of the solutions were then used as template to re-amplify the band fragments using the couple primers 968f-GC/518r, Lac1-f/Lac2-GC andAlfie1-GC/Alfie2-r. Then, the positions of the excised bands in DGGE gel were confirmed with repeated DGGE. Bands showing the expected melting position in DGGE gels were amplified with the couple primers, without GC-clamp and the obtained amplicons were purified (Nucleospin gel and PCR clean-up kit; Macherey-Nagel GmbH & Co. KG, Germany). Finally, purified PCR products were sequenced by a commercial sequencing facility (EurofinsMWG Operon, Ebersberg, Germany). Sequence chromatograms were edited and analysed using the software programs Finch TV version 1.4.0 (Geospiza Inc., Seattle, WA, USA) and identity was then determined by the megablast algorithm in the GenBank database (http://www.ncbi.nlm.nih.gov/BLAST/).

## Abbreviations

FHB: Fusarium head blight
SEM: Scanning electron microscopy
PCR-DGGE: Polymerase chain reaction-denaturing gradient gel electrophoresis
GS: Zadoks growth stage
DI: Disease incidence
DS: Disease severity
LPs: lipopeptides
MRS: de Man, Rogosa and Sharpe broth
NB: Nutrient Broth
PDA: Potato Dextrose agar
